# The Effects of Contact With Nature During Outdoor Environmental Education on Students’ Wellbeing, Connectedness to Nature and Pro-sociality

**DOI:** 10.3389/fpsyg.2021.648458

**Published:** 2021-05-04

**Authors:** Sabine Pirchio, Ylenia Passiatore, Angelo Panno, Maurilio Cipparone, Giuseppe Carrus

**Affiliations:** ^1^Department of Dynamic and Clinical Psychology and Health Studies, Sapienza University of Rome, Rome, Italy; ^2^Experimental Psychology Laboratory, Department of Education, Roma Tre University, Rome, Italy; ^3^Department of Human Science, European University of Rome, Rome, Italy; ^4^University Consortium for Socio-Economic Research and for the Environment (CURSA), Rome, Italy

**Keywords:** environmental education, outdoor, wellbeing, connectedness to nature, pro-sociality

## Abstract

Experiences of contact with nature in school education might be beneficial for promoting ecological lifestyles and the wellbeing of children, families, and teachers. Many theories and empirical evidence on restorative environments, as well as on the foundations of classical pedagogical approaches, recognize the value of the direct experience with natural elements, and the related psychological and educational outcomes (e.g., positive emotions, autonomy, self-efficacy, empathy). In this work we present two studies focusing on the contact with nature in outdoor education interventions with primary and secondary school students in Italy. A questionnaire measuring connectedness to nature, psycho-physical wellbeing, pro-environmental attitudes, students’ life satisfaction, pro-social behavior, empathy and anxiety was completed before and after the education program by the participants to the intervention group and by students of a control group. The students in the intervention groups (154 in study 1 and 170 in study 2) participated in environmental education programs consisting in guided activities in contact with the nature during four visits in one of two natural protected areas. The students in the control groups (253 in study 1 and 168 in study 2) attended the same schools as the intervention group but they were not involved in the environmental education program. The students in both the groups completed the questionnaire in the same weeks of the year. Findings show that taking part to the outdoor education program has positive outcomes on psycho-physical wellbeing, on connectedness to nature and on pro-social behavior of students in the intervention group, compared to the control group. The implications related to the effectiveness of outdoor education interventions and future directions of research and practice in environmental psychology and education are discussed.

## Introduction

Outdoor environmental education programs are a crucial tool for promoting children’s and adolescents’ pro-environmental attitudes and behaviors, as well as their feelings of connection to nature, and pursuing the goal of reducing human impact on the environment and natural resources therein (Passafaro et al., 2010; Pirchio et al., 2020). In the last 30 years, many different approaches have been used and tested to this aim, such as educational programs focusing on the acquisition of knowledge about the environment and how human activities impact on its quality. Other approaches focused on educational experiences where the contact with natural settings and outdoor activities are proposed as a mean for promoting positive emotional reactions among students and facilitating conceptual knowledge of major environmental issues (Rickinson, 2001). Indeed, several studies showed that, to the purpose of an effective behavioral change, educational approaches that are capable to generate an emotional involvement in environmental problems may be more effective than those focusing on the mere knowledge of environmental facts (e.g., Passafaro et al., 2010).

The present study assesses the psychological outcomes of an outdoor environmental education program for primary and lower secondary school students in Italy. Given the extended literature and the solid empirical evidence of the benefits of contact with nature for human psychophysical health and wellbeing, we test the hypothesis that outdoor environmental education programs might not only impact on pro-environmental variables, but also on students’ wellbeing.

In fact, the experience of natural environments has been shown to promote recovery from stressful experiences, allowing individuals to recover their cognitive and emotional resources depleted in the course of daily life tasks, thus helping human adaptation to the environment and promotes subjective wellbeing, as well as physical and mental health (e.g., Hartig, 2004; Nilsson et al., 2010; Marselle et al., 2020, 2021).

Thus, in the present study we explore the impact of contact with nature during an outdoor environmental education program on outcomes relative to both subjective wellbeing (e.g., perceived wellbeing, empathy, anxiety, pro-sociality, and life satisfaction) and to pro-environmental psychological variables (e.g., connectedness to nature, pro-environmental attitudes, and behaviors).

### Outdoor Education and Pro-environmental Outcomes

An important construct to understand the relationship between humans and nature is connectedness to nature, which can be defined as the individuals’ perception of their connection to the non-human natural world (e.g., Mayer and Frantz, 2004; Amerigo et al., 2012; Capaldi et al., 2014). Many studies showed that the perceiving oneself as “connected” to nature is a main predictor of pro-environmental attitudes and behaviors (Mayer and Frantz, 2004; Nisbet et al., 2009; Olivos et al., 2011; Frantz and Mayer, 2014; Pasca et al., 2017); connectedness to nature has therefore been proposed also as a relevant measure for assessing environmental education programs (Frantz and Mayer, 2014; Barrable and Booth, 2020). In fact, contact with nature plays a key role in developing nature connectedness (Nisbet et al., 2009), and those environmental education interventions providing a sustained and emotionally significant contact with nature may increase the perception of being connected to, and part of, the wider natural world among children and adolescents (Barrable and Booth, 2020).

A major aim of outdoor environmental education interventions is to provide students with the opportunity of knowing relevant facts about the ecological processes of natural environments, and to develop positive attitudes and behaviors toward environmental preservation. Most of the studies in the last decades, aiming to explore the outcomes of outdoor environmental education programs, found an effect on environmental knowledge and attitudes (Bogner, 2002; Bogner and Wiseman, 2004; Olivos-Jara et al., 2013; Liefländer and Bogner, 2014; Schmitz and Da Rocha, 2018). Yet, while the role of knowledge in promoting ecological behaviors has been considered as controversial (Vicente-Molina et al., 2013; Moss et al., 2017; Otto and Pensini, 2017), the link between pro-environmental attitudes and ecological behaviors has received greater empirical corroboration (see Kaiser et al., 1999). Outdoor visits to natural spaces, as long as they can provide students with an intense and prolonged positive experience in nature, might thus have an impact on ecological behaviors, together with factual knowledge of, and positive attitudes toward, the natural environment (Bogner, 1998; Dillon et al., 2006; Braun et al., 2018).

### The Impact of Outdoor Education on Well-Being

Starting from the concept of restorative environments, many studies showed the benefits of contact with nature (e.g., in green residential areas, botanical gardens, urban forests, etc.) on human subjective well-being, focusing in particular on adults (Hartig, 2004; Lafortezza et al., 2009; Hartig et al., 2011; Carrus et al., 2015b, 2017; Wassenberg et al., 2015).

Fewer studies have dealt specifically with the experience of restorative environments among children (e.g., Bagot et al., 2015; Carrus et al., 2015a; Collado and Staats, 2016). Children’s activity in natural environments has been associated with cognitive, physical, affective, and moral developmental positive outcomes and with children’s levels of independence and autonomy (Adams and Savahl, 2017). Also, the experience of contact with nature may play a role in attentional processes (Taylor et al., 2001; Johnson et al., 2019; Federico, 2020) and in cognition and emotion functioning among pre-school and school children (Wells and Evans, 2003; Corraliza et al., 2012; Collado et al., 2013; Carrus et al., 2015a). School garden activities and outdoor play have shown a positive effect on children’s self-esteem, wellbeing, and empathy (Dyg and Wistoft, 2018; Sando, 2019). Green life environments (school and residential) may also moderate the impact of stressful life events on children, and improve their physical and mental health (Bell and Dyment, 2008).

In particular, connectedness to nature seems to play an important role in these benefits of contact with nature, being significantly linked to both hedonic and eudaimonic well-being (Bowler et al., 2010; Capaldi et al., 2014; Warber et al., 2015; Whitten et al., 2018).

The main aim of the two studies presented here is to analyze the outcomes of an experience of contact with nature on psycho-physical wellbeing, during a non-residential outdoor environmental education program conducted for children of Primary and Secondary schools in Italy. Students participating in an outdoor environmental education program were matched with students of the same schools which did not take part in the program, as a control group. Thus, both studies analyze the effects of nature experience on an intervention (participating students) and a control (non-participating students) group, before and after the outdoor education program.

The program was composed of several outdoor workshop activities proposed and designed by experts and teachers, involving both the students and their parents during the Spring season. The activities took place during school time, and children were always supervised by their main classroom teachers. The program was supported by the Italian Ministry of Health; the present study received additional support from CURSA (University Consortium for Socio-Economic Research and for the Environment) and from Sapienza and Roma Tre universities.

All students (intervention and control groups) filled out a questionnaire before and after the education program. Data were collected with the agreement of the head teachers, without interfering with the normal organization of the school activities and teachers commitments. For both studies, written informed consent to participation was provided by parents, prior to the data collection.

## Study 1

Study 1 was conducted in the Pantanello natural reserve located in the Lazio Region (about 100 km south of Rome), an area owned and managed by the Fondazione Roffredo Caetani^1^. The environmental education program included three outdoor visits to Pantanello in March, April, and May 2018 for the students and their teachers, plus a fourth final visit where also the parents were involved; the fourth visit was designed with the purpose of letting the parents learn about the workshop experiences directly from the children.

The visits were structured in several educational activities, according to four main workshops carried out in the natural area. The workshops were titled as follows.

(1) “*The plant landscape of the Park: orient yourself among the ancient knowledge on the use of medicinal herbs*”; the children discovered the use of medicinal herbs through the explanations and stories provided by the educators, as well as directly through their sensorial experiences (e.g., colors, smells, tactile experiences). A connection between the experience in nature and school activities was also activated (e.g., children studied in depth the medicinal plants and shared their knowledge with the classmates, created a medicinal plants cookbook, etc.).
(2) “*Healthy as a fish: paths in the Park, to feel good*”; the children were led to discover the park through their own movements, and bodily and motor experiences. The educators designed different psychomotor paths to activate children’s gross and fine motor skills in contact with nature (e.g., jumps, somersaults, etc.). In the class activities, children worked on the connection with the experience in nature; they also designed the paths on paper by exercising their memory and their visual-spatial skills.
(3) “*Biodiverse and… unbalanced: a path to accessibility*”; in this workshop, the activities were designed to accompany children on the search for traces left by wild animals, and to the discovering and recognition of signs of different plant species. Children observed footprints and signs, dens and nests, leaves and seeds, colors and shapes; the skills related to the experience of sounds in nature were also stimulated, in line with many studies pointing on the importance of soundscapes as key component of positive human experiences of nature (e.g., Aletta et al., 2019; Ratcliffe, 2021): children listened to songs, sounds, and noises, to stimulate a wider sensorial perception and understanding of the ecosystems they were experiencing. Moreover, they reasoned with the educators on how humans have transformed the environment. In class, the children discovered with their teachers the natural features and the characteristics of their territory and discussed interventions to safeguard the environment and the pursuit of a more sustainable lifestyle.
(4) “*EcoArt and Map of Emotions*”; the main goal of this workshop was to make the children aware of how they feel and what they think during the experience of nature. Children were led to explore some specific and iconic places of the park, and then asked to report on a map their emotions experienced in these specific places. The work on emotions continued in class. Children studied the characteristics of the emotions, how these can be shared with others, how these can be represented by drawings or by other forms of expression. They were also guided in the realization of art products, which were exposed and shared with the other participants during the outdoor experience.

### Aims and Hypothesis

Our main aim was to analyze the effect of the experience of contact with nature on several variables, during the outdoor program in the intervention group and compare it with the control group, across T1 (March 2018) and T2 (June 2018), and to examine the relations among these variables. Students completed a questionnaire measuring pro-environmental attitudes and behaviors, connectedness to nature, psycho-physical wellbeing, pro-social behaviors, empathy, and student’s life satisfaction before and after the educational program.

We hypothesized that:

– H1: connectedness to nature is positively related to pro-environmental attitudes, psycho-physical wellbeing, pro-social behaviors, empathy, and student’s life satisfaction;
– H2: the experience of contact with nature during the outdoor education program positively influences connectedness to nature, pro-environmental attitudes, psycho-physical wellbeing, pro-social behaviors, empathy, student’s life satisfaction at T2 (after intervention): we expect differences between the scores at T1 and T2 among students in the intervention group compared to the control group.

### Participants

A total of 407 students of six different schools (located near the Pantanello area) participated in the study (54.1% males); 246 students attended the fourth and the last (5th) year of Primary School (age ranged from 9 to 10 years old), and 161 students attended the first year of Junior High School (11 years old). The intervention group was composed by 154 students and the control group by 253 students.

### Instruments

The questionnaire was composed by six scales, measuring the following variables:

1. *Connectedness to nature:* nine items (e.g., “Human beings are part of the natural world”), adapted and translated from the CNS scale (Mayer and Frantz, 2004), to be rated on a 4-steps Likert scale from “completely disagree” (1) to “completely agree” (4).
2. *Psycho-physical wellbeing:* five items with the “how did you feel in the last month” format (e.g., “I felt happy and in a good mood”), to be rated on a 4-steps scale from “never” (1) to “always” (4), taken from the World Health Organization-Five Well-Being Index (WHO-5; Topp et al., 2015).
3. *Pro-social behaviors*: four items regarding individuals’ self-efficacy beliefs, feelings and management of interpersonal relationships (e.g., “if I see someone who is sad, I go to console him”), to be rated on a 3-steps scale from “never” (1) to “many times” (3), taken from the Perceived Social Self-Efficacy Scale (Di Giunta et al., 2010).
4. *Empathy*: three items regarding beliefs on abilities to recognize feelings, emotions and needs of others (e.g., “I understand if my friend needs help even if he doesn’t ask me”), taken from the Perceived Empathic Self-efficacy Scale (Di Giunta et al., 2010), to be rated on a 4-steps scale from “not at all” (1) to “completely” (4).
5. *Student’s life satisfaction*: seven items (e.g., “I like the activities offered at school”), regarding the students’ satisfaction for their school (three items), their living environment (two items), and their self (two items), adapted from Huebner et al. (2012), to be rated on a 4-steps Likert scale from “completely disagree” (1) to “completely agree” (4).
6. *Pro-environmental attitudes and behaviors*: four items adapted and translated from the CATES – Children’s Attitudes Toward the Environment scale by Musser and Malkus (1994) related to children’s pro-environmental action (e.g., “I turn off the water when I brush my teeth”), to be rated on a 4-steps Likert scale from “agree” (1) to “disagree” (4).

### Statistical Analysis

Correlation analysis and repeated measures ANOVA were conducted to test our hypotheses.

### Results

As predicted, (H1), connectedness to nature scores measured at T1 and T2 (as displayed in Table 1) show a positive and significant relation with pro-environmental attitudes and behaviors, with psycho-physical wellbeing, with pro-social behaviors, with empathy and with students’ life satisfaction. Descriptive statistics for all the measures are reported in Table 2.

**TABLE 1:** Bivariate correlations between connectedness to nature and pro-environmental attitudes (PEA), psycho-physical wellbeing (PPW), pro-social behaviors (PSB), empathy (EMP), and student’s life satisfaction (SLS) at T1 (1) and T2 (2).

	PEA1	PPW1	PSB1	EMP1	SLS1	PEA2	PPW2	PSB2	EMP2	SLS2
CN1	0.543*	0.367*	0.361*	0.318*	0.549*	0.519*	0.363*	0.197*	0.228*	0.453*
CN2	0.473*	0.261*	0.268*	0.163*	0.469*	0.507*	0.371*	0.369*	0.375*	0.538*

*p < 0.001.

**TABLE 2:** Descriptive statistics (means and standard deviations) in the intervention and control groups at pre (T1) and post (T2) test, Study #1.

	Group		
Variable	Intervention	Control	Tot
CNS T1	3.44 (0.39)	3.24 (0.51)	3.31 (0.48)
CNS T2	3.49 (0.39)	3.18 (0.56)	3.29 (0.53)
Wellbeing T1	2.98 (0.61)	2.78 (0.52)	2.85 (0.56)
Wellbeing T2	3.21 (0.51)	2.75 (0.58)	2.91 (0.60)
Pro-sociality T1	2.59 (0.28)	2.50 (0.41)	2.53 (0.37)
Pro-sociality T2	2.59 (0.35)	2.51 (0.39)	2.54 (0.37)
Empathy T1	3.14 (0.53)	3.22 (0.57)	3.19 (0.56)
Empathy T2	3.19 (0.51)	3.17 (0.60)	3.18 (0.57)
Life satisfaction T1	3.50 (0.39)	3.24 (0.53)	3.33 (0.50)
Life satisfaction T2	3.46 (0.42)	3.20 (0.53)	3.29 (0.51)
Pro-environmental T1	3.54 (0.48)	3.19 (0.64)	3.31 (0.61)
Pro-environmental T2	3.49 (0.50)	3.23 (0.61)	3.32 (0.59)

*Standard deviations are reported in brackets. CNS, Connectedness to nature scale; Pro-environmental, Pro-environmental attitudes and behaviors.*

**FIGURE 1:**
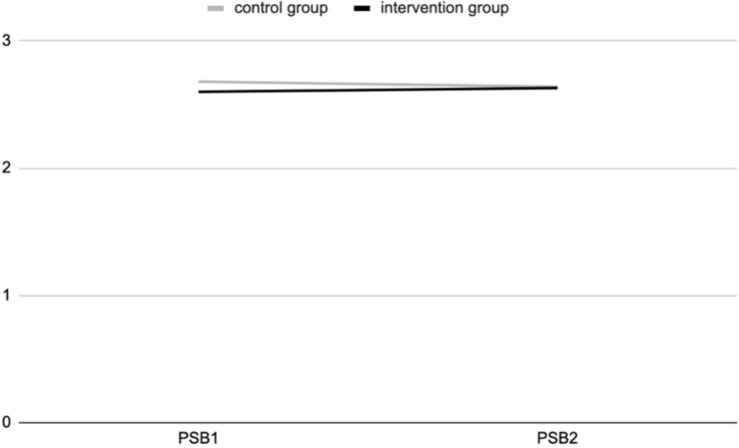
Connectedness to nature (CN) at T1 and T2 for intervention and control groups (study 1).

**FIGURE 2:**
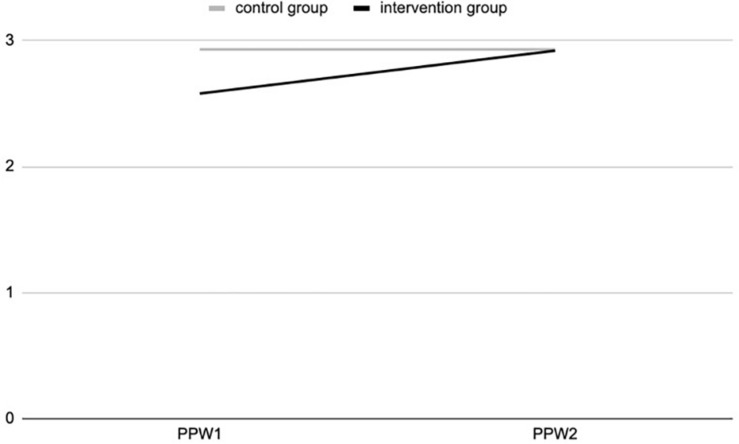
Psycho-physical wellbeing (PPW) at T1 and T2 for intervention and control groups (study 1).

The ANOVA results show a positive and significant effect of the experience of contact with nature during the outdoor education program for the intervention group, at T2, for connectedness to nature [*F*_(311,1)_ = 5.545; *p* = 0.019] (Figure 1) and psycho-physical wellbeing [*F*_(318,1)_ = 16.7; *p* = 0.000] (Figure 2), as a 2-way interaction effect of group (intervention vs. control) by time (pre-post).

No significant interaction effects were observed for pro-environmental attitudes and behaviors, [*F*_(315,1)_ = 2.434; *p* = 0.120], pro-social behaviors [*F*_(317,1)_ = 0.306; *p* = 0.581], empathy [*F*_(3__17__,1)_ = 2.22; *p* = 0.137], and student’s life satisfaction [*F*_(306,1)_ = 0.001; *p* = 0.961].

## Study 2

Study 2 was conducted in part in the same location of study 1 (the Pantanello natural preserve), and in part in a different location: the “Sughereta” of Pomezia, a natural area also located in the Lazio region, about 30 km South of Rome.

The organization of the contact with nature experience during the outdoor education program and the research design was the same as in study 1, and took place from March to May 2019.

The specific activities of the workshops were inspired by the theory of “multiple intelligences” proposed by Gardner (1983). The educational program aimed at stimulating a wide range of capacities in students (such as logic, musical, visual, etc.), through different activities to be carried out in the outdoor green space. The activities were organized around five main workshops, dedicated to the following themes, always designed to improve the students’ mastery and awareness of their own personal skills and competencies in the natural environment: (1) emotions and use of sensorial skills; (2) orienting oneself into the natural setting; (3) narratives and myths related to the natural site and natural elements; (4) biodiversity in the park; (5) psychomotor paths in the park and motor skills through the natural elements. The main principle of the intervention was using the natural setting as a “field laboratory,” promoting the exploration and the physical movement in the park and following the learning goals chosen by the educators and the teachers during the training phase of the project.

### Participants

A total of 338 students of six different schools participated in the study (48.8% males). 171 students attended the fourth and the last year of Primary School (range age from 9 to 10 years old) and 167 students in the first year of Junior High School (11 years old), 170 students were in the intervention group and 168 in the control group. Three schools were located in the area of Pantanello reserve (same as Study 1), and three schools in the area of the Sughereta of Pomezia.

### Instruments and Hypotheses

We used the same questionnaire as in study 1, adding the following measure:

7. *Anxiety*: four items of the SAFA-A (Cianchetti and Sannio Fancello, 2001), assessing self-reported anxiety (e.g., “I worry about the things I have to do”), to be rated on a 4-steps Likert scale from “agree” (1) to “disagree” (4).

The hypotheses were the same as in Study 1, with the addition of anxiety (we expected contact with nature to be negatively linked to anxiety).

### Results

Correlation analyses show a positive significant association of connectedness to nature at T1 and T2 with pro-environmental attitudes, psycho-physical wellbeing, pro-social behaviors, empathy, student’s life satisfaction, and anxiety measured at T1 and T2 (see Table 3). Descriptive statistics for all the measures are reported in Table 4.

**TABLE 3:** Bivariate correlations between connectedness to nature and pro-environmental attitudes (PEA), psycho-physical wellbeing (PPW), pro-social behaviors (PSB), empathy (EMP), school life satisfaction (SLS), and anxiety (ANX) at T1 (1) and T2 (2).

	PEA1	PPW1	PSB1	EMP1	SLS1	ANX1	PEA2	PPW2	PSB2	EMP2	SLS2	ANX2
CN1	0.491*	0.270*	0.335*	0.437*	0.461*	0.104	0.322*	0.212*	0.296*	0.335*	0.349*	0.115*
CN2	0.395*	0.227*	0.315*	0.312*	0.403*	0.109	0.418*	0.327*	0.406*	0.400*	0.471*	0.158*

*p < 0.001.

**TABLE 4:** Descriptive statistics (means and standard deviations) in the intervention and control groups at pre (T1) and post (T2) test, Study #1.

	Group		
Variable	Intervention	Control	Tot
CNS T1	3.27 (0.46)	3.29 (0.46)	3.28 (0.46)
CNS T2	3.28 (0.46)	3.36 (0.48)	3.32 (0.47)
Wellbeing T1	2.59 (0.57)	2.93 (0.51)	2.76 (0.57)
Wellbeing T2	2.92 (0.60)	2.93 (0.56)	2.93 (0.58)
Pro-sociality T1	2.60 (0.40)	2.68 (0.28)	2.64 (0.34)
Pro-sociality T2	2.63 (0.38)	2.64 (0.32)	2.64 (0.35)
Empathy T1	3.10 (0.53)	3.29 (0.47)	3.20 (0.51)
Empathy T2	3.19 (0.53)	3.33 (0.50)	3.26 (0.52)
Life satisfaction T1	3.27 (0.45)	3.36 (0.44)	3.31 (0.45)
Life satisfaction T2	3.22 (0.50)	3.28 (0.51)	3.25 (0.50)
Pro-environmental T1	3.41 (0.48)	3.53 (0.50)	3.47 (0.49)
Pro-environmental T2	3.41 (0.57)	3.50 (0.54)	3.45 (0.55)
Anxiety T1	2.74 (0.70)	2.64 (0.67)	2.69 (0.69)
Anxiety T2	2.80 (0.72)	2.68 (0.76)	2.74 (0.74)

*Standard deviations are reported in brackets. CNS, Connectedness to nature scale; Pro-environmental, Pro-environmental attitudes and behaviors.*

**FIGURE 3:**
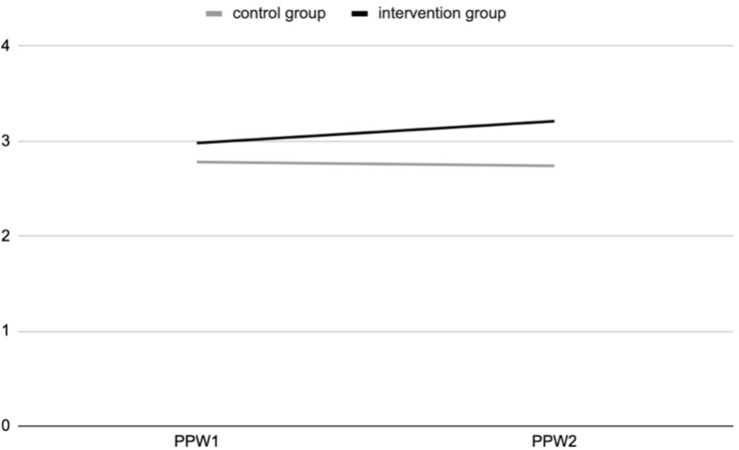
Psycho-physical wellbeing (PPW) at T1 and T2 for intervention and control groups (study 2).

The ANOVA analyses (see Figure 3) show a positive and significant effect of the contact with nature during the outdoor education program for the intervention group, at T2, on psycho-physical wellbeing [*F*_(319,1)_ = 24.428; *p* = 0.000], as a 2-way interaction effect of group (intervention vs. control) by time (pre-post).

**FIGURE 4:**
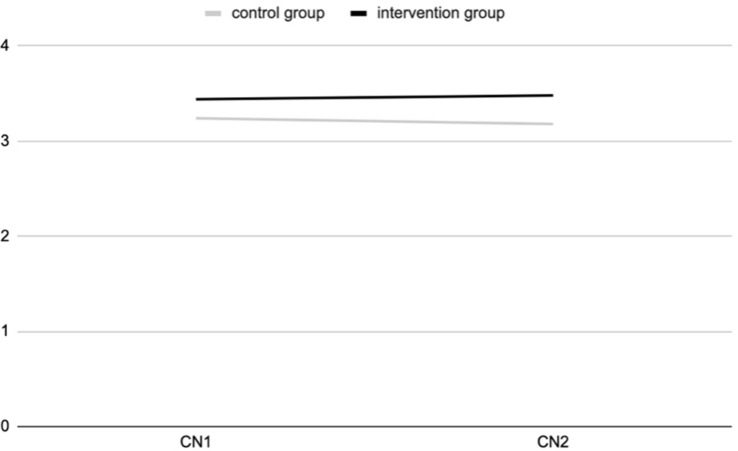
Pro-social behaviors (PSB) at T1 and T2 for intervention and control groups (study 2).

A positive interaction effect, with a tendency to statistical significance, was also observed on pro-social behaviors, increasing at T2 in the intervention group, compared to the control group [*F*_(314,1)_ = 3.225; *p* = 0.073], as reported in Figure 4.

No significant interaction effects were observed on connectedness to nature [*F*_(312,1)_ = 1.738; *p* = 0.188], pro-environmental attitudes [*F*_(309,1)_ = 0.409; *p* = 0.523], empathy [*F*_(314,1)_ = 0.129; *p* = 0.720], student’s life satisfaction [*F*_(310,1)_ = 0.636; *p* = 0.426], and anxiety [*F*_(311,1)_ = 0.316; *p* = 0.462].

## Discussion

This research aimed at describing the outcomes of an experience of contact with nature during school based outdoor environmental education programs, both in terms of students’ pro-environmental orientations (e.g., sense of connection to nature and ecological attitudes and behaviors) and in terms of psychological wellbeing and socio-emotional factors.

Our findings are promising in showing that the participation in the outdoor program, providing contact with natural environments, is associated with higher connectedness to nature, psycho-physical wellbeing, and pro-social behavior in the students of the intervention group, compared to a control group. This is consistent with previous research findings outlining the impact of outdoor educational programs on connectedness to nature (Passafaro et al., 2010; Olivos-Jara et al., 2013; Frantz and Mayer, 2014; Otto and Pensini, 2017; Barrable and Arvanitis, 2018; Barrable, 2019a, b). Also, we observed an association between connectedness to nature and pro-environmental attitudes and behavior, confirming previous findings about the role of connectedness to nature for the development of ecological attitudes and behaviors (Mayer and Frantz, 2004; Nisbet et al., 2009; Frantz and Mayer, 2014). The nature-based interventions investigated in our studies had an impact on subjective wellbeing as well, in both studies, plus on connectedness to nature in the first study, and on pro-sociality in the second study.

We could speculate here on a possible explanation for this differential pattern of findings emerged across studies 1 and 2. In fact, it is interesting to point out the commonalities and differences between the activities performed in the two outdoor educational interventions carried out in the two studies, in line with previous works (Rickinson, 2001). Both the interventions that were assessed in our research targeted factors such as the exploration and motor activities in the natural settings which could have played a role in increasing the students’ perception of wellbeing (e.g., describing the natural landscape or specific plants or animals; making graphic representations of the landscape; performing gross motor actions such as running, climbing, moving using natural elements as tools or barriers, etc.). There were, however, some differences between the two interventions. The educational intervention reported in the first study had a somehow stronger focus on the relations between human beings and nature, strengthening the reflection on how the human activity may modify the natural environment and how nature could be a resource for human activity: in fact, the workshops were designed to guide students in the discovery of activities that can be made with the natural elements, such as the use of medicinal herbs for cooking or coloring, or using natural elements for producing art pieces, or learning how the natural features of the environment can stimulate a wider sensorial perception and understanding of the ecosystems. This could have worked for increasing the students’ reflection on their relation to nature and their sense of connectedness to the natural environment.

These issues were also addressed in the intervention described in study 2, but with a somehow less salient focus. Rather, the intervention conducted in the second study was more related to the development of personal and social skills, based on the general framework provided by the model of multiple intelligences (Gardner, 1983; Gardner and Hatch, 1989). One could argue that that the theoretical framework of the multiple intelligences used for designing the workshops in Study 2 might have led the students to focus more on other aspects of the environmental education experience: for example, the activities in the “social intelligence” domain could be an explanation of the increased pro-sociality scores in the participant students.

Our findings do not show an impact of the outdoor educational program on other hypothesized factors, such as empathy, life satisfaction or anxiety. Even if these variables are correlated with connectedness to nature, suggesting that a sense of belonging to the natural world may in general be linked with positive emotional states and capacities, their scores did not differentiate the intervention and control groups as a direct effect of the contact with nature. This unexpected finding could be explained by the organization of the interventions. In fact, there is evidence of the role of the timing and intensity of the experience of contact with nature for its impact on connectedness to nature and wellbeing. Residential educational programs may have a stronger impact, and longer interventions may be more powerful than shorter ones (Rickinson, 2001; Passafaro et al., 2010; Warber et al., 2015; Barrable and Booth, 2020). The interventions described in this paper included only four visits to the natural settings, lasting a maximum of 4 hours each (which corresponds to a school time morning), about once a month from March to June: such a schedule may not allow for an intense and deep enough experience in nature, capable of making a difference in the intrapersonal variables that were measured here. Clearly, it must be underlined the speculative nature of our explanation: thus, this aspects could be subject to further investigation in the future, by planning and implementing interventions with different levels of length and intensity, in order to involve schools and students in an optimal exposition to natural stimuli.

Finally, the association between connectedness to nature and socio-emotional variables and wellbeing offers interesting indications about the role of contact with nature and outdoor activities in the psychological and subjective wellbeing. As noted in previous studies, a strong sense of connection to nature has the power to increase the positive effect of contact with nature on psychological wellbeing and stress resilience (Mayer et al., 2009), also contributing to increased pro-sociality and empathy (e.g., Whitten et al., 2018). Again, the role of specific outdoor education experiences could be an interesting issue to be addressed by future studies in this field.

In conclusion, in these times where the ecological crisis, the climate emergency and the uncertain and unequal access to natural resources demand a radical change in human behaviors toward the environment and the adoption of more sustainable lifestyles, outdoor education programs targeting the new generations’ environmental knowledge have a major importance (Pugnetti, 2020). Environmental educators need theoretically sound and empirically grounded knowledge, to design effective and efficient intervention programs, in order to impact on participants’ sustainable lifestyles, resilience, and wellbeing (e.g., Warber et al., 2015; Varela-Candamio et al., 2018; Carrus and Panno, 2019; Steinebach and Langer, 2019).

The possibility of promoting more sustainable lifestyles and promoting human resilience by increasing connectedness to nature also through effective education practices is thus a crucial goal and challenge for the advancement of current education systems at the global level.

## Data Availability Statement

The raw data supporting the conclusions of this article will be made available by the authors, without undue reservation.

## Ethics Statement

Ethical review and approval was not required for this study on human participants in accordance with the local legislation and institutional requirements. Written informed consent to participate in this study was provided by the participants’ legal guardian/next of kin.

## Author Contributions

SP and YP planned and implemented the research and wrote and revised the manuscript. SP planned and supervised the data collection. YP performed the statistical analyses and conducted the data collection. GC wrote and revised the manuscript and planned and supervised the data collection. AP supervised the statistical analyses of study 1 and the research design and selection of the instruments. MC was responsible of the organization, planning, and implementation of the educational intervention. All authors contributed to the article and approved the submitted version.

## Conflict of Interest

The authors declare that the research was conducted in the absence of any commercial or financial relationships that could be construed as a potential conflict of interest.

## Footnotes

1 https://www.frcaetani.it/en/pantanello-park/
